# Peroxynitrite and Peroxiredoxin in the Pathogenesis of Experimental Amebic Liver Abscess

**DOI:** 10.1155/2014/324230

**Published:** 2014-04-15

**Authors:** Judith Pacheco-Yepez, Rosa Adriana Jarillo-Luna, Manuel Gutierrez-Meza, Edgar Abarca-Rojano, Bruce Allan Larsen, Rafael Campos-Rodriguez

**Affiliations:** ^1^Sección de Estudios de Posgrado e Investigación, Escuela Superior de Medicina, IPN, Plan de San Luis y Díaz Mirón s/n, 11340 México, DF, Mexico; ^2^Departamento de Morfología, Escuela Superior de Medicina, IPN, Plan de San Luis y Díaz Mirón s/n, 11340 México, DF, Mexico; ^3^Departamento de Bioquimica, Escuela Superior de Medicina, IPN, Plan de San Luis y Díaz Mirón s/n, 11340 México, DF, Mexico

## Abstract

The molecular mechanisms by which *Entamoeba histolytica* causes amebic liver abscess (ALA) are still not fully understood. Amebic mechanisms of adherence and cytotoxic activity are pivotal for amebic survival but apparently do not directly cause liver abscess. Abundant evidence indicates that chronic inflammation (resulting from an inadequate immune response) is probably the main cause of ALA. Reports referring to inflammatory mechanisms of liver damage mention a repertoire of toxic molecules by the immune response (especially nitric oxide and reactive oxygen intermediates) and cytotoxic substances released by neutrophils and macrophages after being lysed by amoebas (e.g., defensins, complement, and proteases). Nevertheless, recent evidence downplays these mechanisms in abscess formation and emphasizes the importance of peroxynitrite (ONOO^−^). It seems that the defense mechanism of amoebas against ONOO^−^, namely, the amebic thioredoxin system (including peroxiredoxin), is superior to that of mammals. The aim of the present text is to define the importance of ONOO^−^ as the main agent of liver abscess formation during amebic invasion, and to explain the superior capacity of amoebas to defend themselves against this toxic agent through the peroxiredoxin and thioredoxin system.

## 1. Introduction

Amoebiasis is a result of infection with the enteropathogen protozoan* Entamoeba histolytica* (*E. histolytica*). Once the amoeba has established itself in the host, its dissemination to the liver and the formation of abscesses in this organ lead to high morbidity and mortality.

Patients with amebic liver abscess (ALA) arrive to the hospital in the chronic phase of this pathogenesis, when the abscess has already formed. Therefore, observations in patients of the first phase of ALA, involving the inflammatory reaction of the immune response, have been sporadic (when patients arrive for other reasons and inflammation is found). In order to understand the first phase of ALA in humans, animal models have been employed. These models have proven useful even though there are important differences between hamsters and humans in the chronic phase of ALA [1, 2].

The molecular mechanisms by which* E. histolytica* causes ALA are still not fully understood. Based on evidence from the hamster model of amoebiasis, it seems that an inadequate immune response in individuals susceptible to amoebiasis fails to impede an amebic invasion, thus leading to uncontrolled inflammation. Chronic inflammation is accompanied by the production of several toxic molecules, including nitric oxide (NO) and reactive oxygen intermediates (ROIs), such as the superoxide anion (O_2_^−^). Recently it has become clear that these molecules are substrates for synthesis of a highly oxidizing agent known as peroxynitrite (ONOO^−^). Furthermore, reports have pointed out that* E. histolytica* has very effective defense mechanisms against the formation of ONOO^−^, as well as for its interception and inactivation. The principal mechanism for ONOO^−^ interception seems to be the thioredoxin system. The aim of present text is to define the importance of ONOO^−^ as the main agent of liver abscess formation during amebic invasion and to explain the apparently superior capacity of the amoeba to defend itself against this toxic agent through the peroxiredoxin and thioredoxin system.

## 2. Adhesion and Cytotoxic Activity of Amoebas during Amoebiasis and ALA

There is still sharp controversy among scientists today regarding the molecular mechanisms of amoebiasis and amebic liver invasion caused by the pathogen* E. histolytica*. Some researchers pose that the direct damage to liver tissue by amoebas (through mechanisms of adherence and toxicity) causes the formation of liver abscess during amoebiasis. Other researchers assert that the importance of amebic mechanisms in the initial stage of amoebiasis is to allow the pathogens to survive and multiply in microenvironments, thus provoking the chronic inflammatory response that provides the principal mechanisms of damage to liver tissue, eventually resulting in ALA. To a great extent, the controversy hinges on an ambiguous use of language on both sides of the question.

Adherence of amoebas to the colonic epithelium and other host cells is unquestionably of fundamental importance in amoebiasis during the initial stages of the pathogenesis, which include the initial infection and extraintestinal invasion. That is, adherence is essential for the amoeba to invade the host and establishes itself in microenvironments in which it can survive. The virulence factor of adherence is to a great extent mediated by the N-acetyl-D-galactosamine inhibitable (Gal/GalNAc) lectin [3, 4], the 220 kDa lectin [5], and other adhesins such as the 112 kDa adhesin [6]. There are reports of reduced amebic adherence to human erythrocytes, neutrophils, colonic mucins, and epithelia when amebic lectins are inhibited [5–7]. Recently it was shown that EhCPADH, a complex formed by a cysteine proteinase and an adhesin, mediates adherence, phagocytosis, and cytolysis. Immunization with a recombinant polypeptide, EhADH243, induces protection in hamsters against development of ALA [8]. It has also been demonstrated that the recombinant enzyme rEhCP112 digested gelatin, collagen type I, fibronectin, hemoglobin, and Madin-Darby canine kidney (MDCK) cell monolayers; the EhCP112 enzyme is secreted by the trophozoites [9].

The Gal/GalNAc lectin is a virulence factor required for trophozoite adherence to target cells, as shown by inhibition of this lectin with galactose [10, 11]; cell killing occurs in the typical Gal/GalNAc manner. It has been shown that peroxiredoxin (Prx) interacts with Gal/GalNAc lectin and that the lectin-peroxiredoxin complex is located at the amoeba-host cell contact sites. Therefore, the recruitment of Prx probably protects the trophozoites against the ROS generated by host cells (phagocytic and epithelial), which would facilitate the invasion [12, 13].

Molecular mechanisms of pathogenicity that allow* E. histolytica* trophozoites to survive and proliferate within a wide variety of host environments are related not only to adherence but also to cytotoxic activity. Trophozoites can damage host cells through direct contact or close proximity, as well as through phagocytic activity towards dead and dying host cells in a receptor-mediated fashion [14]. Of the molecules secreted by amoebas, cysteine proteases are particularly important [15–20]. They are responsible for a cytolytic effect on host cells [7], the modulation of the cell-mediated immune response, and the proteolysis of the host extracellular matrix [16, 21–25]. Previous works show that when mutated, cysteine proteinase 5 (CP5) has a reduced activity and the trophozoite has a reduced ability to generate ALA [26].

Extensive tissue damage has been attributed to the cysteine proteinases of* E. histolytica* because they are (i) secreted in large quantities and can cleave extracellular matrix proteins, thus facilitating amebic invasion; (ii) secreted in higher quantities by virulent than nonvirulent trophozoites; and (iii) found to participate in the inflammation of the gut and ALA [15–20, 27]. However, cysteine proteases are dispensable for phagocytosis and cytopathogenicity [28, 29]. Their main physiological role could be the digestion of host cells, as they are required for rosette formation, hemolysis, and digestion of erythrocytes.

Another molecule that could participate in the damage of host cell is the cyclooxygenase- (COX-) like enzyme in* E. histolytica*, responsible for the biosynthesis of prostaglandin E_2_ (PGE_2_). The production of PGE_2_ by the COX-like enzyme in amebic liver granuloma can downregulate effector and accessory cell functions of infiltrate immune cells [30].

Although the aforementioned processes are fundamental in the initial stages of amoebiasis, they seem to be quite secondary as mechanisms of necrosis leading to hepatic abscesses in the latter stages of this disorder. Adhesion is important for the survival of amoebas and the establishment of prolonged contact of amoebas and toxic molecules with host endothelial cells. However, neither adhesion nor these toxic molecules seem to be responsible for the host tissue damage that directly results in amebic colitis and ALA. These disorders occur during the latter stages of amoebiasis, when the majority of host cells in contact with amoebas appear not to be damaged [31–33]. It seems likely that damage to host cells at this time is carried out in function of the inflammatory process [31].

The proposal that host cell damage results mainly from a chronic inflammatory response is corroborated by a number of different studies. Some recent reports [34, 35] are revealing in this sense, as they analyze the pathogenesis of ALA in hamsters inoculated with engineered HGL-2 trophozoites defective in Gal/GalNAc function. The authors show that HGL-2 amoebas infect hamster liver despite lacking this important adhesion molecule, although the pattern of infection is different from that produced by wild-type* E. histolytica*. HGL-2 amoebas cause a large number of inflammatory foci with a disorganized structure, and these foci are located in the vicinity of blood vessels. Despite their reduced capacity for adhering to the host endothelium and penetrating liver tissue [34], these defective trophozoites are able to provoke ALA [35].

In histopathological terms, the chronic phase of ALA in humans corresponds to lytic or liquefactive necrosis, whereas in rodent models there is granulomatous inflammation [1, 2, 36–38]. Hence, hepatic damage in hamsters is caused by apoptosis and necrosis rather than the lytic necrosis found in humans [39–41]. However, hamster models have provided important insights into the possible mechanisms of the inflammatory response to amebic invasion in the acute phase [1, 2].

In a hamster model and at thirty minutes following inoculation,* E. histolytica* amoebas were found in the portal vein (resulting in slightly dilated sinusoids), in the lumen of small branches of the portal vein, and in the central veins [2]. After one hour trophozoites were located in the sinusoids throughout the hepatic lobules [2]. At three hours after inoculation, polymorphonuclear leukocytes (PMNs) surrounded the amoebas with one or several layers of cells and thus impeded them from making direct contact with hepatocytes. Nonetheless, lysis continued. Under these conditions, the leukocytes that managed to make direct contact with amoebas were undamaged [2]. Afterwards, there was a continual increase in the quantity of PMNs and the lysis of leukocytes in liver sections during the development of ALA. The massive destruction of leukocytes favored greater necrosis and hemorrhaging of parenchymal tissue and the formation of ischemic areas. Whereas few amoebas were found in necrotic areas at this stage, there are many in the periphery of the lesion, where mononuclear cells (histiocytes with an epithelioid appearance) started to form a palisade that separated parenchymatous cells from the necrotic area.

Since studies with hamster models demonstrate that ALAs generally develop in the absence of direct contact with amoebas, it is necessary to explore the possible mechanisms that could account for their formation. The mechanisms of chronic inflammation seem to be a likely candidate [2, 42, 43]. Of course, it is the adhesion of amoebas and their capacity to evade the host immune defenses that provoke a continuous inflammatory response [31, 44–46].

## 3. Chronic Inflammation as the Possible Cause of ALA

### 3.1. Nitric Oxide and the Pathogenesis of ALA

Some research groups have long suspected that chronic inflammation is the cause of ALA. Until recently, the mechanisms of chronic inflammation considered as the cause of abscess formation were mainly the amebicidal effects of NO, ROIs, cytokines, and cysteine proteinases [45, 47–51].

In this sense, it has been emphasized that greater quantities of NO are found in the serum of hamsters with liver abscesses than in healthy animals [52, 53]. Some reports have shown that the amebicidal activity of activated macrophages is mainly associated with NO synthesis [54–56]. Nevertheless, the amoebas continue to survive and proliferate.

Some studies have reported that reactive oxygen species (ROS) and NO produced by activated neutrophils or macrophages lead to the lysing of* E. histolytica* [56–60].* In vitro* studies used a high concentration of NO (1 mM) [48, 54, 61, 62], whereas* in vivo* the concentration of NO in inflamed tissues is approximately 1 *μ*M [60, 63]. During the development of ALA, there is evidence of trophozoite resistance to high concentrations of NO* in vitro *[48] and* in vivo* [52, 53]. Despite the relatively high iNOS mRNA expression and NO production,* E. histolytica* continues to show an invasive capacity* in vivo*. Hence, the percentages of amoebas that remain viable represent a sufficient number to sustain an amebic invasion.

Two hypotheses can be formulated based on the apparent lack of effectiveness of NO as an amebicidal agent* in vivo*: (i) that NO is not really toxic enough to carry out an effective amebicidal function or (ii) that virulent amoebas have an adequate defense mechanism against NO activity. Regarding the latter conclusion, several studies indicate that the capacity of* E. histolytica* to resist the destructive action of NO and ROIs, whether* in vivo* or* in vitro*, probably owes itself to the expression by this* Entamoeba* species of high levels of antioxidant proteins, such as Prx, flavoprotein A, superoxide dismutase (SOD), and rubrerythrin [64–67].

### 3.2. The Possible Role of Peroxynitrite in Inflammation and ALA

It is now known that there are molecules of the inflammatory response much more detrimental than NO. In fact, NO is a precursor of one of these molecules. During the inflammatory process, NO and the O_2_^−^ are produced simultaneously, and they react at diffusion-controlled rates to produce the ONOO^−^ anion [63, 68]. The reaction is as follows:

(1) $$\begin{array}{c} \text{NO}+{{\text{O}}_{2}}^{-}⟶{\text{ONOO}}^{-} \end{array}$$

Unlike NO and O_2_^−^, ONOO^−^ is not a free radical, but it is a highly oxidizing agent. The coupling of NO with O_2_^−^ to yield ONOO^−^ in biological systems is currently accepted as the main biological source of ONOO^−^, which is extremely reactive with biological molecules and highly toxic to cells.

There is no direct* in vivo* evidence of ONOO^−^, probably due to its high reactivity [69, 70]. However, nitrate and nitrite, the products of spontaneous decomposition of this molecule, have been detected in the serum of hamsters with ALA [52, 53].

**Figure 1:**
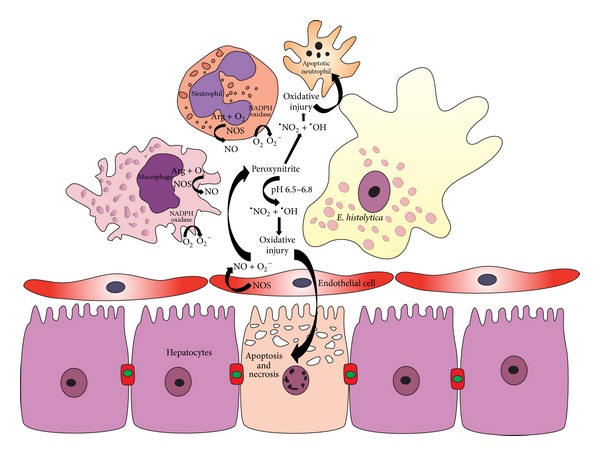
Hypothetical model of the generation of ONOO^−^ in ALA. Under inflammatory conditions the two substrates of ONOO^−^, nitric oxide (NO) and the superoxide anion (O_2_^−^), are produced simultaneously. This allows for the production of large quantities of ONOO^−^, which is a highly oxidizing agent. Indeed, the products of spontaneous decomposition of ONOO^−^, nitrate and nitrite, are found at higher than normal levels in inflamed tissue. The superoxide anion is produced by NADPH oxidase expressed in activated neutrophils, macrophages, and endothelial cells. NO is produced by NOS found in inflammatory cells or in the vascular endothelium. The protonated form of ONOO^−^ is disintegrated to form free nitrogen dioxide (^•^NO_2_) and the hydroxyl radical (^•^OH). Under conditions of hepatic hypoxia, low concentrations of L-arginine and oxygen enhance arginase activity that hydrolyzes L-arginine, thus competing with the substrate for NOS enzymes. This leads to the uncoupling of NOS, simultaneously producing O_2_^−^ and NO, and thus resulting in additional ONOO^−^ generation. ONOO^−^ reacts directly with thiol groups of host cells. High levels of ONOO^−^ lead to the development of ALA by inducing apoptosis and necrosis in hepatic cells.

The two substrates of ONOO^−^, NO and the O_2_^−^, seem to be abundantly produced in inflammatory sites during the host response to amebic invasion [31, 52, 53, 71]. For instance, O_2_^−^ is produced by the reaction of NADPH oxidase (or xanthine oxidase) and dioxygen (O_2_) (Figure 1) via electron leakage in the mitochondrial respiratory chain and at the endoplasmic reticulum [70]. The levels of O_2_^−^ can undergo a 3- to 4-fold increase during the inflammatory response when the NADPH oxidase complex expressed in phagocytic cells and endothelial cells is activated and reacts in the oxygenated environment to generate O_2_^−^ (Figure 1) [68, 72, 73].

To some extent,* in vitro* and* in vivo* reports are contradictory. On the one hand,* in vitro* studies report that macrophages isolated from ALA in gerbils are deficient in their capacity to produce NO as a result of the modulation of iNOS mRNA [74]. On the other hand, there are studies using a hamster model that report the* in vivo* production of NO in the zone of liver abscess, mediated by iNOS mRNA expression [53]. The deficient capacity of macrophages isolated from ALA for developing a respiratory burst does not denote the absence of the O_2_^−^ anion, because the production of this anion does not depend solely on this mechanism.

The production of NO requires the presence of a nitric oxide synthase (NOS), a family of enzymes that includes iNOS, eNOS, and nNOS. iNOS can be found in the inflammatory cells, while eNOS is present in the vascular endothelium [63, 75]. In sites of inflammation, the cytokine-induced activity of iNOS increases. The iNOS-mediated formation of O_2_ via a novel pathway in L-Arg depletes murine macrophages, and the O_2_ formed in this way interacts with NO to form ONOO^−^, which then enhances macrophage immune function. However, the overproduction of these oxidants could also trigger cell death (Figure 1) [76].

Both NADPH oxidase and NOS are found in phagocytes and activated NOS is found in endothelial cells (Figure 1) [69]. Although NO is a relatively stable and highly diffusible free radical, O_2_^−^ is very short lived and has restricted diffusion across biomembranes. Thus, the presence of O_2_^−^ would seem to be the limiting factor for the synthesis of ONOO^−^. Nutrient deprivation, ischemia, and cytokines (e.g., TNF*α* and IL-1) under the hypoxic conditions of inflammation all increase the activity of endothelial NADPH oxidase and the consequent production of O_2_^−^ [72].

Under some inflammatory conditions the production of NO and O_2_^−^ is strongly activated, which could lead to a 1,000-fold increase in the production of each. This in turn would cause a 1,000,000-fold increase in the formation of ONOO^−^ [63, 69, 70]. High levels of ONOO^−^ lead to the dysfunction of critical cellular processes, the disruption of cell signaling pathways, and the induction of cell death through both apoptosis and necrosis [63]. Thus, ONOO^−^ is thought to be the potential cause of a number of inflammatory diseases [68] and could possibly lead to the development of ALA.

The production of ONOO^−^ is increased by two mechanisms under conditions of ischemia or hypoxia. Firstly, hypoxia in the liver leads to low concentrations of L-arginine and oxygen (hypoxia and anoxia), which enhance macrophage arginase activity [77–80]. Arginase, the principal enzyme of the urea cycle, hydrolyzes L-arginine to urea and L-ornithine. On the other hand, L-arginine and a cofactor (tetrahydrobiopterin, BH4) are substrates for NOS enzymes. Thus, arginase competes with NOS enzymes for their common substrate (L-arginine), leading to the uncoupling of all three isoforms of NOS [80].

Secondly, hypoxia induces production of the hypoxia inducible factor, which also promotes the uncoupling of NOS [81–83] as well as the activation of arginase-2 [84]. This uncoupling simultaneously produces O_2_^−^ and NO [85], leading to additional ONOO^−^ generation [69, 70, 76, 86]. Indeed, more ONOO^−^ is formed in hypoxic regions of the liver where the pH is low, leading to the production of hydroxyl-like free radical species in the absence of oxygen [87].

ONOO^−^ is capable of reacting directly or indirectly with biological tissues. It reacts directly with thiol groups, the preferential targets for ONOO^−^ reactivity* in vivo* [70], causing oxidative damage to iron-sulfur centers and the active site of -SH groups in tyrosine phosphatases, lipids, and CO_2_. On the other hand, ONOO^−^ can act indirectly by decomposing into highly reactive radicals. The ONOO^−^ ion is more stable than its protonated form (ONOOH, pKa 6.5 to 6.8), which decomposes rapidly via homolysis of the O–O bond to form free nitrogen dioxide (^•^NO_2_) and the hydroxyl (^•^OH) radical (Figure 3). The latter radical has a highly oxidizing effect on many biomolecules, including tyrosine residues, thiols, DNA, and unsaturated fatty-acid-containing phospholipids [68, 88].

Compared to the reactivity of the ^•^NO_2_ and ^•^OH radicals, the reactions of ONOO^−^ are relatively slow. Thus, ONOO^−^ should certainly be more selective for its target molecules and better able to react relatively far from its site of formation [70]. When generated from a cellular source, ONOO^−^ could influence surrounding target cells within one to two cellular layers (~5–20 *μ*m) [69].

By reacting with lipids, DNA, and proteins via direct oxidative reactions or via indirect radical-mediated mechanisms, ONOO^−^ can trigger cellular responses ranging from subtle modulations of cell signaling to overwhelming oxidative injury that commits cells to necrosis or apoptosis [69]. When damage mediated by ONOO^−^ induces cell necrosis, the release of the intracellular content of cells (e.g., HMGB1: high mobility group protein B1) into the extracellular space can trigger an additional inflammatory response, representing a positive-feedback cycle of further ONOO^−^ generation [69].

DNA damage induced by ONOO^−^, leading to the activation of poly (ADP-ribose) polymerase (PARP), can also increase the inflammatory response. It has been shown that PARP coactivates many inflammatory cascades and increases tissue infiltration by activated phagocytes in experimental models of inflammation, circulatory shock, and ischemia-reperfusion [63, 89]. Through these mechanisms, ONOO^−^ amplifies neutrophil-dependent responses (adhesion, migration, and activation of neutrophils) under inflammatory conditions, and thus contributes to the detrimental effects of inflammation in arthritis, colitis, and other inflammatory diseases [63, 70].

## 4. Defense Mechanisms against ONOO^−^

In mammalian tissue, peroxides like H_2_O_2_ and ONOO^−^ are produced as a result of normal cellular processes, including metabolism and inflammation. As a result, host tissue and amoebas must have defense mechanisms against these molecules. To be able to provoke an uncontrolled inflammatory response, the trophozoite defense system against ONOO^−^ would have to be better than that of the host. Biological protection against ONOO^−^ is organized in two main categories: prevention and interception [90, 91].

### 4.1. Prevention of ONOO^−^ Formation

One defense mechanism by amoebas and mammalian tissue against ONOO^−^ is the prevention of its formation through the inhibition of its precursors, NO and O_2_^−^ [92]. The steady state concentrations of O_2_^−^ are normally kept relatively low, in the nanomolar to picomolar range. The main route of O_2_^−^ consumption in biological systems is its reaction with superoxide dismutase (SOD) to form hydrogen peroxide (H_2_O_2_) and dioxygen. On the other hand, NO is scavenged by hemoglobin, decreasing its levels in the blood [93]. NO readily reacts with oxyhemoglobin or oxymyoglobin to give nitrate (NO_3_^−^) and oxidized hemoproteins (methemoglobin and metmyoglobin) as follows:

(2) $$\begin{array}{c} \text{Hb}\left(\text{Fe}-{\text{O}}_{2}\right)+\text{NO}⟶\text{metHb}\left(\text{Fe}\;\;\text{III}\right)+{\text{N}{\text{O}}_{3}}^{-} \end{array}$$

Due to the high concentrations of oxyhemoglobin in the body, this molecule may provide the primary metabolic as well as detoxification mechanism for NO* in vivo* [93–95]. Thus, red blood cell-encapsulated hemoglobin can react very quickly with NO and scavenge virtually all the molecules produced by endothelial and inflammatory cells [94, 95]. However, the inactivation of NO by Hb and by self-oxidation could be less efficient in a hypoxic environment, as this reaction requires oxygen.

One generally recognized mechanism for the inactivation of NO is its reaction with O_2_ [93]. Since NO and O_2_ are much more soluble in lipid layers than aqueous fractions, biological membranes may act as a “lens” that can focus and magnify the self-oxidation of NO [96, 97].

*E. histolytica* has developed some mechanisms to avoid NO production. For example, soluble amoeba proteins suppress INF*γ* induced amebicidal activity, thus affecting the expression of the mRNA iNOS gene and consequently NO production [74]. Moreover, there is evidence that the monocyte locomotion inhibitory factor of* E. histolytica* inhibits the* in vitro* NO production normally induced by cytokines in human leukocytes [98]. It has also been reported that* E. histolytica* arginase inhibits NO production by consuming L-arginine, the substrate of iNOS in activated macrophages. All of these amebic mechanisms should certainly contribute to the survival* E. histolytica* [99].

### 4.2. Interception or Inactivation of ONOO^−^

Due to a direct reaction with low-molecular weight compounds (e.g., carbon dioxide, thiols, ascorbate, selenocompounds, and synthetic metalloporphyrins) and other proteins (e.g., certain peroxidases, hemoglobin, albumin, and selenoproteins), ONOO^−^ is decomposed into nontoxic products. Efficient ONOO^−^ scavengers include selenium compounds, particularly 2-phenyl-1, 2 benzisoselenazol-3(2H)-one (ebselen), selenomethionine, and selenocysteine, as well as selenium-containing proteins, such as glutathione peroxidase and thioredoxin-reductase [70, 90]. Additionally, (−)-epicatechin and other flavonoids can contribute to the cellular defense against ONOO^−^ [91]. Considering their approximate concentrations* in vivo* and their reaction rate constants in relation to ONOO^−^, the best of these low-weight molecules for the interception of ONOO^−^ would seem to be CO_2_, hemoglobin, and glutathione [90].

## 5. The Thioredoxin System

The principal mechanism for interception of ONOO^−^ is probably the thioredoxin system, which is comprised of thioredoxin (Trx) and thioredoxin reductase (TrxR). This system together with peroxiredoxin (Prx) mediates the NADPH-dependent reduction of H_2_O_2_ and tert-butyl hydroperoxide in* Entamoeba* species [100]. For Trx to carry out most of its functions, the disulfide active site in this protein must be reduced in the following manner:

(3) $$\begin{array}{c} \text{Oxidized}\;\;\text{Trx}+\text{TrxR}\;\;\text{(with}\;\;\text{NADPH)}⟶\text{Dithiol}\;\;\text{Trx} \end{array}$$

Once Trx accepts electrons from TrxR, it can reduce Prx. Another NADH enzyme, flavin oxidoreductase, also carries out this function [100]. The resulting form of Prx can protect against oxidative stress by decomposing H_2_O_2_ into water (H_2_O) and probably ONOO^−^ into nitrite [101].

### 5.1. TrxR

The different functions of Trx are entirely dependent upon the activity of TrxR [102], and the latter has two major forms in nature. Whereas the larger form of TrxR has a selenoprotein (~Mr = 55 kDa) and corresponds to mammals, the relatively small nonselenoprotein form of TrxR is found in bacteria, plants, archaea, and most unicellular eukaryotes [102, 103].

Homodimeric mammalian TrxR consists of two subunits in a head-to-tail arrangement. Both subunits are absolutely required for normal catalysis during the catalytic cycle [102]. The C-terminal -Gly-Cys-Sec-Gly-COOH motif of the human selenoprotein (about 16 amino acids long) is found in all mammalian TrxR, and the Cys-Sec dyad of this motif has been identified as a reversible selenenylsulfide/selenothiol, constituting the active site of the enzyme [102]. The electrons from NADPH reduce a redox active disulfide and transfer them to the C-terminal active site of a selenothiol located in the sequence Gly-Cys-Sec-Gly, which is conserved in all isoforms of TrxR. From this site electrons are transferred to Trx, allowing the latter to carry out its functions, such as the reduction of protein disulfides or other substrates [104].

For example, the two major forms of human Trx, cytosolic Trx1 (the TXN1 gene product) and mitochondrial Trx2 (the TXN2 gene product), are reduced by TrxR and NADPH [101, 102]. As a result, these two forms of Trx can provide electrons to proteins such as ribonucleotide reductase (formation of deoxyribonucleotides from ribonucleotides). In this way Trx reacts with ribonucleotide precursors to synthesize deoxyribonucleotides, peroxiredoxins, and methionine sulfoxide reductases [102].

As can be appreciated, the Trx-reducing activity of mammalian TrxR is totally selenium dependent [102], and aberrations in selenium metabolism must have a direct impact on the functions and levels of different selenoproteins, as well as on many cellular systems that are linked to Trx activity.

### 5.2. The Amebic Trx System

**Figure 2:**
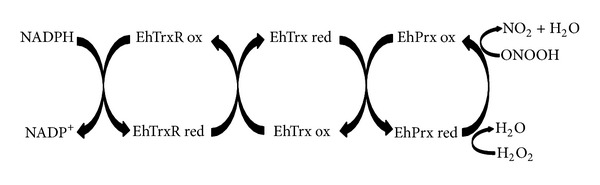
* E. histolytica* thioredoxin system. The* Entamoeba* thioredoxin system is comprised of thioredoxin (Trx) and thioredoxin reductase (TrxR), coupled with peroxiredoxin (Prx) and NADPH. Trx has to be reduced to fulfill its functions, and this reduction is carried out through catalyzation by TrxR using NADPH as a cofactor. Once Trx accepts electrons from TrxR, it can reduce peroxiredoxin (Prx). The resulting form of Prx can protect against oxidative stress by decomposing H_2_O_2_ into H_2_O. Based on the figure by Arias et al. [105].

Previous works reported the molecular cloning, expression, purification, and functional characterization of two genes from* E. histolytica* that encode for TrxR and Trx (EhTrxR and EhTrx). It turns out that EhTrxR belongs to the low-molecular-weight family of TrxRs, which have a redox active site containing two key cysteine residues without a selenoprotein [100]. This is a homodimeric covalent protein that catalyzes the NAD(P)H-dependent reduction of thioredoxins (Figure 2) and S-nitrosothiols [92].

Unlike the TrxR of* E. histolytica*, that of mammals is dependent on selenoproteins. On the other hand, both mammalian TrxR and EhTrxR reduce the corresponding form of Trx by using NADPH [100]. The resulting form of Trx reduces Prx, which in turn can reduce H_2_O_2_ to H_2_O and probably ONOOH to nitrite (Figure 2), thus acting as an antioxidant system [100].

EhTrxR, EhTrx, and Prx have been immunolocalized under the plasma membrane in* E. histolytica*. Contrarily, mammalian TrxR, Trx, and Prx have not been found under the membrane (see Section 5.3.4) [64, 105]. This cellular location in* E. histolytica* favors the potential* in vivo* functionality of the ROS detoxification system. On the one hand, one amebic mechanism of resistance to lipid peroxidation is the composition of the amebic membrane, which has a high concentration of saturated lipids [106]. On the other hand, the EhTrx system could offer an alternative protective mechanism against lipid peroxidation, thus maintaining intracellular proteins and DNA safe from highly toxic reactive oxygen and nitrogen species. In such a case, this would represent a key mechanism in relation to the virulence of* E. histolytica* when exposed to highly toxic ROIs [105] and should be pivotal to amebic survival during extraintestinal infection [92].

### 5.3. Peroxiredoxin

Peroxiredoxins (Prxs) are a family of antioxidant enzymes that are principally reduced by Trx [107]. Once reduced, Prx can in turn reduce H_2_O_2_ and perhaps ONOO^−^ to harmless forms. These peroxide-eliminating enzymes are truly ubiquitous, existing in yeast, plant, and animal cells, including protozoa, helminths, parasites, and most (if not all) eubacteria and archaea [108]. Prxs are preferentially expressed under conditions of stress induced by elevated levels of reactive oxygen species (ROS) and reactive nitrogen species.

#### 5.3.1. Mammalian Prx

There are at least five subfamilies of mammalian Prx enzymes, which are categorized on the basis of their protein sequence and cellular location. Although located primarily in the cytosol, Prx is also found within mitochondria, chloroplasts, and peroxisomes, associated with nuclei and membranes [108, 109]. The two categories of Prx consist of 1-Cys and 2-Cys. Each form has a distinct number of cysteinyl residues involved in catalysis [108, 109].

#### 5.3.2. Amebic Prx

Although parasites are in an anaerobic environment during colonization of the colon, when invading tissue they are exposed to aerobic conditions. Several studies have identified H_2_O_2_ as lethal to* Entamoeba* species [57, 58, 64, 110]. Thus, in the oxygen-rich microenvironment that exists at the site of hepatic lesions, the capacity of Prx to prevent the deleterious production of H_2_O_2_ in the host must certainly play an important role in the survival of parasites [65, 111–113].

*E. histolytica* Prx (EhPrx) can degrade H_2_O_2_ [111, 114] as well as alkyl and aryl hydroperoxides [64]. It may also degrade ONOO^−^ to nitrite. Like mammalian Prx, the peroxidase activity of EhPrx depends on Trx and TrxR activity [115]. EhPrx removes H_2_O_2_ *in vitro* in the presence of NADPH, EhTrxR, and EhTrx (Figure 2). This capacity of Prx has been confirmed in both native and recombinant proteins [111, 114, 115].

Virulent* E. histolytica* is reportedly more resistant to H_2_O_2_ than* E. dispar* and the nonvirulent strains of* E. histolytica* [64, 116, 117]. There are reports of an increase in protein and gene expression of Prx by* E. histolytica* in an oxygen-rich environment [65, 112]. By employing a fluorometric stopped-flow assay, the H_2_O_2_ detoxification activity of EhPrx and Prx of* E. dispar* was found to be similar [64]. Thus, it seems that the principal difference between EhPrx and the Prx of* E. dispar* is the location of this antioxidant. Accordingly, the ability of* E. dispar* to become virulent under certain conditions may be due in part to the possible change of location of Prx in distinct environments [114]. Trophozoites deficient in Prx are more sensitive than the wild type to an oxygenated environment and to H_2_O_2_ in axenic culture [118].

Logically, the size of liver abscesses is lower in hamsters inoculated with Prx-downregulated* E. histolytica* trophozoites compared to normal HM1:IMSS (6–9 versus 20–25 mm) [118]. Similarly, overexpression of Prx in* E. histolytica* Rahman rendered the transgenic trophozoites more resistant to killing by H_2_O_2_ (5 mM)* in vitro* [116] and Rahman trophozoites expressing higher levels of Prx have been associated, based on histological analysis of human colonic xenografts, with higher levels of intestinal inflammation and more severe disease [116].

Trophozoites cultured axenically are less virulent, both in the hamster model of ALA and in the mouse model of amebic colitis [119, 120]. RNA from such low virulence trophozoites was compared with the transcripts of amoebas whose virulence was maintained by passing them through hamster liver abscesses. Among the upregulated transcripts found in the virulent amoebas were those involved in both oxidative and stress defense, including three Prx transcripts [121]. Hence, Prx levels of* E. histolytica* HM1:IMSS trophozoites may increase in response to the presence of host inflammatory cells [116].

Moreover, immunization of gerbils and hamsters with recombinant Prx results in partial protection against ALA formation and/or a reduction in the size of the abscesses [122–124]. Thus a strengthened immune response against amebic Prx may protect hamsters against the development of ALA [123].

The evidence regarding the activity and location of EhPrx, together with the resistance provided by Prx to the toxic effects of H_2_O_2_, clearly suggests the importance of this antioxidant enzyme for the survival and virulence of* E. histolytica* trophozoites [116, 118].

#### 5.3.3. Prx Structure and Action Mechanisms

EhPrx has a 52% identity with the human PrxII-2 Cys, the main difference being the extended cysteine-rich N-terminal region in the amebic version, probably contributing to its enzymatic activity [111]. Several authors have demonstrated that peroxiredoxin forms a 60 kDa dimer through disulfide bonds [113, 124] and forms an oligomer or oligomers with high-molecular mass on the cell surface (>200 kDa) [112]. Thus, EhPrx is typically found as an obligate homodimer (2-Cys Prxs) with identical active sites [108].

Furthermore, EhPrx can form a high-molecular mass oligomer on the cell surface [111, 112] because it contains two conserved sequence motifs (region I, sequence PLDWTF, and region II, sequence DSVYCHQAWCEA) that are necessary for decamer formation in 2-Cys Prxs [108, 125]. This decameric structure (ring-like higher oligomers or toroids) is comprised of five dimers (that may or may not be functionally active) linked end-on through predominantly hydrophobic interactions [109, 126].

EhPrx retains arginine 163 and the two conserved cysteine residues (VCP motifs, Cys 87 and 208) that are required for its catalytic activity [108, 109, 115]. The structure and sequence of the peroxidatic active site is highly conserved among Prx classes (1-Cys, typical 2-Cys, and atypical 2-Cys Prxs). The features of typical 2-Cys Prx are highly conserved across all kingdoms, with 30% or greater sequence identity. The most conserved regions include (i) the peroxidative cysteine (generally near residue 50) within the DFTFVCPTEI motif in mammals and the DWTFVCPTEI motif in* E. histolytica *and (ii) the resolving cysteine (near residue 170) within the VCP motif [108]. Therefore, the catalytic activity of the Prx of* Entamoeba *spp. must be similar to other typical Cys-2 Prx.

**Figure 3:**
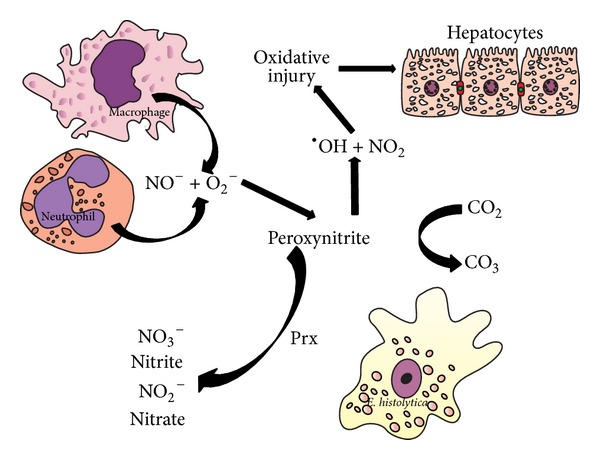
Amebic peroxiredoxin in ALA. During the development of ALA, activated phagocytes produce increased levels of superoxide (O_2_^−^) and NO, thus generating the ONOO^−^ anion, which in turn causes oxidative damage to hepatocytes. ONOO^−^ can decompose into nitrogen dioxide (^•^NO_2_) and the highly oxidizing hydroxyl radical (^•^OH). The ONOO^−^ anion can be intercepted by direct reaction with carbon dioxide, decomposing it into nontoxic products. The Prx enzymes of parasites (e.g.,* E. histolytica*) and other bacteria are involved in protection of these organisms by the reduction of host-produced hydrogen peroxides and ONOO^−^.

Various studies have demonstrated that Prx, whether of mammalian, bacterial, or parasitic origin, can reduce ONOO^−^ to nitrite* in vitro* [69, 127–130]. One study confirmed a protective role of Prx against ONOO^−^ *in vivo* (Figure 3) [101].

#### 5.3.4. Location of Prx

Mammalian Prx is mainly located in the cytosol, membrane, and mitochondria [108]. Contrarily, EhPrx has been detected not only in the nucleus and cytoplasm [131] but also on the peripheral membrane [12, 132, 133]. EhPrx probably forms a high-molecular-mass oligomer (>200 kDa) on the cell surface [111, 112]. Indeed, peroxiredoxin (29 kDa) is the major thiol-containing surface protein of* E*.* histolytica* and has both peroxidase and antioxidant activities [64, 132].

Prx interacts with galactose and the GalNAc lectin, which are both found at sites of amoeba-host cell contact. The recruitment of Prx at this site probably protects trophozoites against the ROIs generated by host phagocytic and epithelial cells [12, 13]. The membrane location of EhPrx suggests an important role for the Trx-dependent metabolic pathway as a redox interchanger, which could be critical for the maintenance and virulence of the parasite when exposed to highly toxic ROIs [105]. In this location the system protects the membrane against lipid peroxidation and may participate in the protection of hemoglobin and other intracellular proteins against free radical damage by stimulating potassium efflux.

The enzymatic activity, quantity, and location of EhPrx probably account for the greater resistance of this amebic strain to oxidants like H_2_O_2_. Hence, the Trx-Prx system of* E. histolytica* seems to help it survive and proliferate in a highly oxygenated environment, leading to greater invasiveness [114] and virulence.

## 6. Other Defense Mechanisms of* E. histolytica* during Amebic Invasion

### 6.1. Flavodiiron Proteins

Flavodiiron proteins are enzymes found in strict and facultative anaerobic bacteria and archaea, as well as a limited number of eukaryotes such as the pathogenic protozoa* Trichomonas vaginalis*,* Entamoeba*, and* Giardia intestinalis *[66, 134–138]. Due to the role of flavodiiron proteins in the catalysis or reduction of O_2_ to H_2_O and NO to N_2_O, they have been proposed as a protective mechanism against nitrosative stress or oxygen toxicity in anaerobes [137, 139, 140].* E. histolytica* has four copies of flavoprotein A [66], suggesting that this enzyme plays a significant role in these two mechanisms. However, the substrate preferences (O_2_ or NO) of the* E. histolytica* flavodiiron proteins and their role during infection have not been determined [141].

### 6.2. Erythrophagocytosis

Phagocytosis is considered a virulence sign in* E. histolytica *[14, 142–145]. Recent data suggest that transmembrane kinases (TMK) have a role in phagocytosis of human erythrocytes [146]. Erythrophagocytosis may be important for the survival, growth, and proliferation of* E. histolytica* in an aerobic or even microaerobic environment [147, 148].* E. histolytica* trophozoites preferentially interact with red blood cells, meaning that the phagocytic activity of erythrocytes by* E. histolytica* trophozoites should certainly have a very important function in amoebiasis. Indeed, the phagocytic capacity of trophozoites has been taken as a qualitative marker of pathogenicity [142, 145, 149]. Erythrophagocytosis may help the amoeba to scavenge NO and other reactive oxygen and nitrogen intermediates and thus help the parasite to survive in the oxygen-rich environment of oxidative stress [147].

Hemoglobin and Prx are two major proteins present in erythrocytes. For example, Prx2 is an abundant erythrocyte protein [109, 147]. Therefore, the process of erythrophagocytosis may serve two important functions in the amebic trophozoites that would contribute to parasite virulence. Firstly, erythrocytes rich in hemoglobin (Hb) may help trophozoites to scavenge NO, which readily reacts with oxyhemoglobin and oxymyoglobin to give nitrate (NO_3_^−^) and the oxidized hemoproteins, methemoglobin, and metmyoglobin. The reaction with hemoglobin is the primary detoxification mechanism for NO [93–95]. Secondly, erythrocytes* rich* in Prx may help amoebas to scavenge ONOO^−^. Hb and Prx-2 of the erythrocytes are extremely efficient at scavenging H_2_O_2_, NO, and ONOO^−^.

These mechanisms are probably pivotal for parasite survival under the nitrogen and oxygen-rich environment of inflammation (Figure 3) [148]. Also, phagocytosis of bacteria containing Prx could increase the pathogenicity of* E. histolytica* in the host intestine and possibly also act as a stimulus to induce the invasive behavior of trophozoites [150].* E. histolytica* might have a mechanism of incorporating Prx similar to that of* Plasmodium falciparum*, which imports Prx-2 from human erythrocytes into its cytosol. This Prx-2 exists in a functional form and a significant concentration [151].

## 7. An Overview of the Prx Model of Amoebiasis

Abundant evidence in the literature suggests that inflammation and inflammatory mediators are the cause of tissue damage in ALA (see Sections 3.1 and 3.2), as few trophozoites are present in the extensive areas of apoptosis and necrosis [152]. Furthermore, in the absence of inflammatory cells, virulent* E. histolytica* trophozoites lead to little or no abscess formation in hamsters [44, 153].

N-Acetylcysteine (NAC) also inhibits tissue damage in the latter stages of ALA in hamster. In the liver of hamsters treated with NAC, there are many clusters of well-preserved amoebas in close contact with hepatocytes, sparse inflammatory infiltrate, and minimal or no tissue damage [42]. The inhibitory effect of NAC on tissue damage during late stages of EALAH was explained [42] as the inhibition of leukocyte-endothelium adhesion molecules and reduced migration of leukocytes, leading to an inhibitory effect of NAC on NO production and ROS activity, and consequently a reduction of the toxic effect on cells. According to Olivos-García et al. [42] “*ROS and NOS (NO and ONOO* ^−^ *) may be the principal molecules responsible for tissue destruction during the late stages of ALA*” [42]. However, NAC also reduces ONOO^−^ mediated toxicity in various pathophysiological conditions [69, 154, 155]. Hence, the point of view in the aforementioned quote is perhaps better explained by the Prx model, which holds that the inhibition of the synthesis of ONOO^−^ would not only maintain amoebas alive but also reduce both inflammatory infiltrate and tissue damage.

### 7.1. Initial Phase: Acute Inflammation

It is well known that some individuals are susceptible to* E. histolytica* trophozoites while others are resistant [31, 156]. In susceptible individuals, trophozoites invade the intestine, survive the host immune response, and arrive to the liver through the blood flow. Their presence in the sinusoids is associated with an influx of host immune cells. We propose that the key to susceptibility is the incapacity of the immune response to eliminate the amoebas before the latter are able to establish colonies and provoke a chronic inflammatory response.

**Figure 4:**
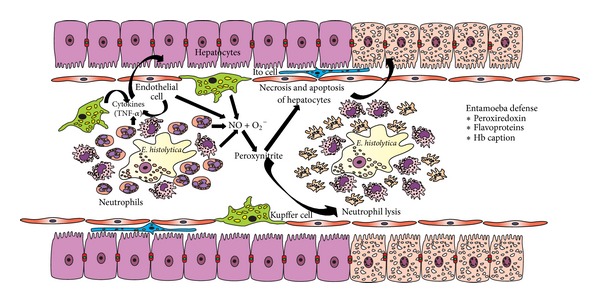
Role of ONOO^−^ and* E. histolytica* Prx in the pathogenesis of ALA. The early stages of ALA are characterized by an acute and chronic host inflammatory response, where an abundant number of monocytes, macrophages, Kupffer cells and activated endothelial cells produce large amounts of nitric oxide and the superoxide anion. Moreover, these molecules may be released after immune cells are lysed. Therefore, there is probably a high concentration of ONOO^−^ at inflammatory sites, which in turn would promote necrosis and/or apoptosis of hepatocytes. Amebic flavoproteins and Prx play a protective role against the oxidative and nitrosative attack of phagocytic cells and against the H_2_O_2_ produced by the amebic metabolism.

During this initial stage of amoebiasis in hamsters, the two major components of the inflammatory response are PMNs and mononuclear cells (MO), both involved in cellular infiltration [1, 2]. PMNs cause the formation of numerous inflammatory foci that produce large amounts of NO and O_2_^−^, which in turn lead to the production of ONOO^−^ (Figure 4). This highly reactive oxidizing agent probably lyses neutrophils, endothelial cells, hepatocytes, and other parenchymal cells. Lysed neutrophils release proteolytic enzymes, adding to the assortment of toxic compounds that cause damage to endothelial, parenchymal, and inflammatory cells (Figure 4) [1, 2]. It has been proposed that with inflammation there is massive trophozoite death [157], which does indeed seem to be the case for individuals resistant to* E. histolytica*. However, for susceptible individuals, it seems more likely that amoebas take advantage of the inflammatory environment in the first stage of the pathogenesis by ingesting nutrients from dead inflammatory cells.

### 7.2. Chronic Phase: Uncontrolled Inflammation

Amoebic lesions in hamsters are formed by necrosis and apoptosis of hepatocytes [1, 2, 37, 39, 40, 152, 157]. The evidence outlined in the present review seems to indicate that these abscesses are not formed principally through direct contact between amoebas and host cells but instead by the uncontrolled inflammatory response produced when susceptible individuals are unable to eliminate amoebas.

Under conditions of uncontrolled inflammation, there is an ever greater quantity of amoebas, amebic molecules (cysteine proteinases, LPFG, Gal/GalNAc-specific lectin, and prostaglandin E2), and cytokines (IL-1a, IL-6, IL-8, and TNF-a) that induce the production of adhesion molecules (e.g., ICAM-1 and E-selectin), which in turn attract macrophages and lymphocytes [31]. These cells together with Kupffer cells produce large amounts of cytokines, which further amplify the inflammatory response [31, 158–160]. The abundant number of monocytes, macrophages, and Kupffer cells, as well as activated endothelial cells and host mitochondria, produce ever greater quantities of NO and O_2_^−^, which probably lead to the formation of large amounts of the ONOO^−^ anion (Figure 4).

The Prx model proposes that diffusible molecules, such as ONOO^−^ and TNF-*α*, are able to commit cells to necrosis or apoptosis in the liver (Figure 4) [68, 69]. Corroborating this idea, one study reported that inflammation is induced when parasites or immune cells secrete or release diffusible products after cell death [51]. ONOO^−^ interacts with lipids, DNA, and proteins via direct oxidative reactions or indirect, radical-mediated mechanisms. Among the latter mechanisms, nitrogen dioxide (^•^NO_2_) and hydroxyl radicals (^•^OH) are of particular importance (Figure 3).

The continuous, prolonged, and uncontrolled inflammatory response seems to be responsible for the production of large granulomas or “abscesses” in hamsters, which are characteristic of the chronic phase of ALA in experimental animal models [31]. At 3-4 days after inoculation of axenic amoebas, the chronic granulomatous reaction in the liver is formed by typical multiple granulomas, which have a central necrosis limited peripherally by a palisade of epithelioid cells and more externally by connective fibers. Amoebas are recognizable between the necrosis and epithelioid cells [1, 2].

With chronic inflammation and necrosis comes ischemia of the lesions [31, 153, 161]. Consequently, there is reduced availability of the host molecules that could counteract ONOO^−^ (e.g., carbon dioxide, hemoglobin, and albumin) [69, 70]. Indeed, it has been shown that in ischemic tissues there is a greater ONOO^−^ concentration [87].

### 7.3. Defense against ONOO^−^: Amoebas versus Host Cells

To be able to provoke an uncontrolled inflammatory response, the* E. histolytica* defense system against ONOO^−^ would have to be better than that of the host. According to evidence in the literature, there are four important factors in this sense.

Firstly, since Prx-2 is an abundant erythrocyte protein [109, 147],* E. histolytica* trophozoites may ingest high quantities of Prx through erythrocyte phagocytosis, as occurs with agents of other parasitic diseases [151]. This would not be the case for host cells. Hence, the quantity of this enzyme in amoebas is probably greater than in mammals. Moreover, erythrocytes, which are rich in hemoglobin (Hb), may help the amoeba to scavenge NO.

Secondly, in* E. histolytica* Prx has a prominent surface and subcortical distribution [12, 131–133], which probably makes these trophozoites more capable of surviving the attack of ONOO^−^ produced externally. Contrarily, Prx is mainly located in the cytosol and mitochondria of hamsters.

Thirdly, the structure of mammalian cells leaves them more susceptible to an ONOO^−^ attack. That is, mitochondria are found in mammalian cells but not in trophozoites, and mitochondria play an essential role in the mechanisms of cell death (by apoptosis and necrosis) triggered by ONOO^−^ [63].

Fourthly, the effectiveness of Prx in mammals is completely selenium dependent, and plasma selenium concentration and the synthesis of selenoproteins (probably including TrxR) seem to be markedly reduced during inflammation, evidenced by a severe drop in the concentration of these proteins in the liver during sepsis or sepsis-like illness [162, 163]. In addition, if indeed selenium inhibits the activation of NF-kappaB, as has been reported [164], the deficit of selenium would tend to feed uncontrolled inflammation, which would further reduce the levels of selenium in liver and serum.

Most ingested selenium enters specific selenium metabolism pathways in the liver, thus providing the raw material for the synthesis of selenoproteins. One such protein produced in the liver is selenoprotein P (SelP), whose main function is the transport of selenium to remote tissues. Additionally, locally expressed SelP may have other functions, as demonstrated in the brain [163, 165]. One of these functions is the chelation of heavy metals, presumably by forming nontoxic selenium-metal complexes, thus preventing cell toxicity and protecting against ONOO^−^ mediated oxidation and nitration [165]. A recent report indicated that SelP protects mice against trypanosomiasis [166]. Therefore the reduction of selenium metabolism and selenoprotein synthesis by hepatic damage should certainly diminish the synthesis and activity of mammalian TrxR in hepatocytes and inflammatory cells.

The apparent importance of the scarcity of selenium in the liver during the pathogenesis of ALA is further supported by the effect of supplementation with selenium during chronic inflammation. The consequence is the restoration of depleted hepatic and serum selenium levels caused by an increase in selenoprotein biosynthesis, leading to the suppression of CRP production and an attenuation of the inflammatory process [164]. However, selenium supplementation can also produce undesirable effects, such as increasing susceptibility to infection by reducing inflammation, or inducing sexually dimorphic effects [167]. The relationship between selenium, selenoprotein synthesis, and host-pathogen interactions is an important issue and warrants further investigation [168].

Hence, EhPrx is considered to be an important protective mechanism against two elements: (i) the oxidative attack by host phagocytic cells activated during the amebic invasion and (ii) the H_2_O_2_ produced by amebic metabolism [114]. Ample evidence indicates that the protection afforded by Prx against ONOO^−^ is essential not only for the survival but also for the pathogenicity of trophozoites.

## 8. Drug Design

The Trx system provides interesting drug design targets. The structural and mechanistic differences, between human and* P. falciparum* TrxR and more generally between the mammalian and the bacterial TrxR, should make this protein a new drug design target for antibacterial agents [103, 165, 169]. Additionally, the stimulation of host or the inhibition of amebic Prx might be a useful strategy for the treatment of amoebic liver abscess.

This area of research is only beginning to be explored. Recently it has been reported that auranofin, a gold-containing drug that inhibits TrxR, has a higher amebicidal activity than metronidazole* in vitro*. In a hamster model of amebic liver abscess, orally administered auranofin markedly decreased the number of parasites, the detrimental host inflammatory response, and hepatic damage. However, many trophozoites are resistant to auranofin [170].

Selenocysteine 496 of human TrxR is a major target of the antirheumatic gold-containing drug auranofin. Although auranofin is a potent inhibitor of mammalian TrxR* in vitro*, it is practically ineffective* in vivo* [171–173].

## 9. Perspective

Amoebiasis caused by* E. histolytica* is an important public health problem in emerging countries. It is generally the poorest who have the greatest exposure to this amebic species and at the same time the greatest susceptibility. Therefore, it is important to understand the pathogenic mechanisms of this disease in order to define molecular targets and design economical drugs to act against such targets.

The hypothesis of the “uncontrolled inflammatory response” as the main cause of amoebiasis has been evolving for approximately three decades. The understanding of the mechanisms of chronic inflammation and their possible relation to amebic colitis and ALA may be reaching the proper depth of understanding in order to be able to finally produce novel therapies. Intervention in the Trx system of the host or parasite could possibly provide the long-sought solution to this public health problem. In relation to* E. histolytica* trophozoites, the surface and subcortical distribution of Prx, which provides this amebic species with many advantages in terms of survival and virulence, might also provide an easily accessible drug target.
